# Biology, ecology, and epidemiology of *Alternaria* species affecting tomato: ground information for the development of a predictive model

**DOI:** 10.3389/fpls.2024.1430965

**Published:** 2024-09-20

**Authors:** Irene Salotti, Paola Giorni, Paola Battilani

**Affiliations:** Department of Sustainable Crop Production (DI.PRO.VES.), Università Cattolica del Sacro Cuore, Piacenza, Italy

**Keywords:** *Alternaria*, tomato, early blight, fruit rot, systematic literature review, modeling

## Abstract

Among pathogens that affect tomato, *Alternaria* spp. are important due to their implications in yield losses and the contamination of tomato products by mycotoxins. In this study, a systematic literature review was conducted to retrieve and analyze available data on the *Alternaria–*tomato pathosystem, with particular attention focused on the main biological processes included in the pathogen life cycle and mycotoxin production. We considered 110 papers (selected from initial 2,138 papers) on five *Alternaria* species that were historically related or recently identified to cause damage to tomato leafage and fruits. Published mathematical models related to *Alternaria* diseases in tomato were also screened based on their aim and development methods, highlighting the wide use of empirical approach. Retrieved information was also evaluated for applications in building a mechanistic, weather-driven model that incorporates the key steps of the pathogen life cycle. This systematic review highlights several knowledge gaps, including the effect of wetness on infection and environmental requirements for mycotoxin production, and suggests paths for further research especially for recently isolated species.

## 1 Introduction

Tomato (*Solanum lycopersicum* L.) is one of the most widely grown vegetables in the world. It is estimated that 186.1 million tons of tomato fruits were produced in 2022 over a harvested area of 4.9 million ha (FAO, 2024). Fresh and processed tomato is an important part of the human diet due to its wide usage and richness in amino acids, sugars, dietary fibers, and minerals (Bergougnoux, 2014). Along the crop value chain, however, various fungal pathogens and spoilage microorganisms may reduce tomato yield and quality. Species belonging to the genus *Alternaria* can infect tomatoes and originate destructive epidemics in the field, particularly early blight and rots (Chaerani and Voorrips, 2006) and postharvest decay (Troncoso-Rojas and Tiznado-Hernández, 2014). Yield losses may be up to 50-80% under favourable weather conditions (Chaerani and Voorrips, 2006; Pandey et al., 2003). Furthermore, *Alternaria* spp. is well-known for its ability to produce a wide range of secondary metabolites, including plant-pathogenic phytotoxins and mycotoxins that can contaminate food products with health risks for humans (Lou et al., 2013; Patriarca, 2016).

The genus *Alternaria* (sub-division *Deuteromycotina*, class *Hyphomycetes*, family *Dematiaceae*) was first established by Nees Von Esenbeck (1817) and has since undergone several revisions, leading to the recognition of 27 sections based on morphology and molecular phylogeny (Lawrence et al., 2016). *Alternaria* species mainly associated with tomato diseases belong to both the small-spored *Alternaria* section, as *A. alternata*, *A. arborescens*, *A. tenuissima*, and *A. tomato*, and the large-spored *Porri* section, as *A. solani* (Bertuzzi et al., 2021; Chaerani and Voorrips, 2006; Habib et al., 2021; Morris et al., 2000; Pose et al., 2010; Sanzani et al., 2019; Somma et al., 2011; Vaquera et al., 2014). These species reproduce asexually, with the asexual spores mainly responsible for the development of epidemics (Lawrence et al., 2016). Adhikari et al. (2021) however reported high genetic variation and genotypic diversity in *Alternaria* populations collected from tomato and potato in North Carolina and Wisconsin, suggesting that sexual reproduction may occur and play an important role in the evolution of these pathogens.

In the field, *Alternaria* infections may occur on all the aboveground components of the plant, leading to different symptoms that can be distinguished as early blight on foliage, fruit rot, collar rot on seedling stems, and stem lesions or cankers in adult plants (Walker, 1952). The initial symptoms of early blight are small, dark, necrotic lesions on older leaves that enlarge at the later stage to form concentric rings, with a target-like appearance, and surrounded by a chlorotic zone (Sherf and MacNab, 1986). Both green and ripe fruits are susceptible to infections that can result in dark, velvety, sunken spots. In some cases, these spots originate from mycelia extending from stem lesions and develop as concentric rings (Sherf and MacNab, 1986). Dark and sunken lesions may appear on seedlings or stems and brunches of adult plants, causing the death of the former and the damage and breaking of the latter (Sherf and MacNab, 1986; Walker, 1952).

Epidemics in the field are primarily initiated by asexual spores (conidia) produced on overwintering inoculum sources, such as crop debris (Jones et al., 1991) or long-lasting spores (e.g., chlamydospores; Basu, 1971; Patterson, 1991). Under favorable temperature and moisture conditions, spores produce one or more germ-tubes that penetrate the host epidermal cells directly via appressoria or enter through stomata or wounds (Sherf and MacNab, 1986). Enzymes and toxins (both host-specific and non-specific) facilitate the colonization of the inner tissue and nutrient extraction and play an important role in plant pathogenesis (Meena and Samal, 2019; Tsuge et al., 2013). The onset of symptoms and sporulation depends on the *Alternaria* species, environmental conditions, plant organ, and host age and resistance (Malathrakis, 1983; Nightingale and Ramsey, 1936; Waggoner and Horsfall, 1969). The secondary spreading of the disease is achieved by conidia produced within lesions and dispersed by wind or occasional rain splashes (Gottwald et al., 1997; Jambhulkar et al., 2016; Pearson and Hall, 1975).

Toxins produced by *Alternaria* species during epidemics accumulate within plant tissues and can contaminate fruits (Lou et al., 2013). Certain species produce several mycotoxins in infected plants (Logrieco et al., 2009) that belong to three different structural groups: dibenzopyrone derivates such as alternariol (AOH) and alternariol monomethyl ether (AME); perylene derivates such as altertoxins (ATX); and tetramic acid derivatives such as tenuazonic acid (TeA) (Pose et al., 2010). Several metabolites are produced by a single *Alternaria* species, while others are produced by more than one species. The most widespread metabolite is alternariol, which has been isolated from different *Alternaria* fungi (Lou et al., 2013). Many of these toxins, including alternariol (AOH), AME, TeA, tentoxin (TEN), and altertoxins (ATX), pose a danger to human and animal health (EFSA, 2011; EFSA et al., 2016). These toxic compounds cause mutagenic, estrogenic, and clastogenic effects in microbial and mammalian cell systems (EFSA, 2011). Surveys performed in several European and South American countries reported that TeA and AOH are found frequently in retail tomato products (Ackermann et al., 2011; Bertuzzi et al., 2021; da Motta and Valente Soares, 2001; Noser et al., 2011; Terminiello et al., 2006). For example, the main contaminant observed in Belgium, Swiss, Brazilian, and Argentinian tomato products is TeA, with an incidence ranging from 18% to 100% (22–85 samples) and TeA concentrations from a few ng/kg to up to more than 4000 μg/kg. AME and AOH were also present in these products, but at a lower frequency or concentration (da Motta and Valente Soares, 2001; Bertuzzi et al., 2021; Noser et al., 2011; Terminiello et al., 2006; Walravens et al., 2016). In contrast, AOH was observed at a high frequency and with a detection limit of 1–13 μg/kg in German tomato products (93% of samples; Ackermann et al., 2011). Several European studies have reported the presence of conjugated mycotoxins such as AOH-3-sulfate and AME-3-sulfate as well as native forms (Puntscher et al., 2019; Walravens et al., 2016). However, the role of many *Alternaria* compounds is still not fully understood. In particular, the toxicological data available in the literature are limited to TeA, AOH, AME, and perylene-derivative altertoxins (ATXs). Even if *Alternaria* species can produce many more metabolites, there are no reports on their function, toxicity, and whether they can be produced in plants (Patriarca, 2016). Only TEN has been identified as a phytotoxin causing chlorosis in the seedlings of numerous plants (da Cruz Cabral et al., 2016).

Despite the increasing attention focused on *Alternaria* mycotoxins both in research programs and risk assessment studies, the proper regulation and legal limits on the maximum concentration of *Alternaria* toxins in tomato-derived products are still lacking. The European Food Safety Authority (EFSA) performed a risk assessment for the main *Alternaria* mycotoxins (AOH, AME, and TeA) and established the threshold for toxicological concern (Solfrizzo, 2017). Moreover, in 2022, the European Union delivered Recommendation (EU) 2022/553, setting the limits for *Alternaria* toxins in several foodstuff, including processed tomatoes (EU Commission, 2022). For example, 5, 10, and 500 μg/kg are the recommended limits for AOH, AME, and TeA in processed tomato products, respectively.

The demand to minimize yield losses while ensuring food safety and agricultural sustainability has resulted in the development of mathematical models to describe or predict the relationships between pathogens, crops, and the environment that trigger epidemics. Such models, which have been developed since the 1950s, are either empirical or mechanistic-based. The former are based on rules or statistical analysis that relate a single or a few components of the disease cycles (e.g., infection, sporulation, or mycotoxin production) with the concomitant weather conditions. The latter are process-based models that exploit pathogen ecology, biology, and epidemiology to represent pathogen disease cycles and disease progress (Battilani, 2016; Rossi et al., 2010).

To improve the understanding and prediction of *Alternaria* diseases and reduce mycotoxin contamination in food and feed, a comprehensive knowledge of ecological conditions that enhance *Alternaria* spp. growth and metabolism in tomato is required. In this review, we focus on the *Alternaria–*tomato pathosystem with the objectives to: (i) synthesize the available literature regarding the biology, ecology, and epidemiology of *Alternaria* spp. affecting tomato; (ii) identify the main knowledge gaps; (iii) determine the similarities and differences between these *Alternaria* spp; (iv) screen published plant disease modes for the *Alternaria–*tomato pathosystem; and (iv) evaluate available information for the development of a mechanistic, weather-driven model to support decision-making in the control of *Alternaria* diseases.

## 2 Methods

### 2.1 Systematic literature search

A systematic literature review was performed to retrieve the available knowledge on three main thematic blocks, namely, the effect of the environment on the main epidemiological components of the *Alternaria* spp. life cycle, the production of mycotoxins, and the published plant disease models for *Alternaria* species. The main epidemiological components considered in this search were: (i) primary inoculum sources; (ii) dispersal of conidia; (iii) germination of conidia; (iv) infection by conidia; (v) mycelial growth; (vi) symptom development; and (vii) production of secondary inoculum.

A protocol was adopted following Okoli and Schabram (2010) for retrieving published papers that contain data of interest for the development of the database. The papers included in the study had to: (i) contain both the terms *Alternaria* and tomato in the title, abstract, and/or keywords; (ii) contain studies conducted within tomato fields or with *Alternaria* strains isolated from infected tomatoes; (iii) contain original data on the effect of the environment on the *Alternaria* spp. life cycle, or on mycotoxin production, or models for *Alternaria* diseases; and (iv) be published in journals, proceedings, or as other forms from competent authorities/organizations. To search the literature, specific queries were formulated based on these criteria for each thematic block. The block concerning the *Alternaria* spp. life cycle was further divided into sub-blocks to better address the search for each epidemiological component (**Table 1**).

**Table 1:** Search strings for biological processes considered for the literature search in three databases and the corresponding number of papers found in each database (N), the total number of papers after the merge or N and removal of duplicates, and the selected number of papers (Sel.).

Biological process	Database	Search query	N	Tot	Sel.
Primary inoculum sources and production	Web of Science	TS=((Alternaria) AND (“Solanum lycopersicum” OR tomato) AND (“primary inoculum” OR “primary inoculum source*” OR overwinter*))	4	4	3
	Scopus	TITLE-ABS-KEY((Alternaria) AND (“Solanum lycopersicum” OR tomato) AND (“primary inoculum” OR “primary inoculum source*” OR overwinter*))	0		
	CAB Ab.	((overwinter$ or primary inoculum) and alternaria and tomato).af.	4		
Dispersal of conidia	Web of Science	TS=((Alternaria) AND (“Solanum lycopersicum” OR tomato) AND (conidia OR spore*) AND (dispersal OR release OR liberation OR dissemination))	18	37	4
	Scopus	TITLE-ABS-KEY((Alternaria) AND (“Solanum lycopersicum” OR tomato) AND (conidia OR spore*) AND (dispersal OR release OR liberation OR dissemination))	7		
	CAB Ab.	((dispersal or dissemination or release or liberation) and Alternaria and tomato).af.	26		
Germination of conidia	Web of Science	TS=((Alternaria) AND (“Solanum lycopersicum” OR tomato) AND (conidia OR spore*) AND (germination OR “germ tube”))	151	254	10
	Scopus	TITLE-ABS-KEY ((Alternaria) AND (“Solanum lycopersicum” OR tomato) AND (conidia OR spore*) AND (germination OR “germ tube”))	94		
	CAB Ab.	((germination or germ tube) and Alternaria and tomato).af.	227		
Infection by conidia	Web of Science	TS=((Alternaria) AND (“Solanum lycopersicum “OR tomato) AND (conidia OR spore*) AND (infect*))	285	489	8
	Scopus	TITLE-ABS-KEY((Alternaria) AND (“Solanum lycopersicum “OR tomato) AND (conidia OR spore*) AND (infect*))	74		
	CAB Ab.	((tomato or solanum lycopersicum) and Alternaria and infection).af.	394		
Mycelial growth	Web of Science	TS=((Alternaria) AND (“Solanum lycopersicum” OR tomato) AND (“mycelial growth” OR invasion OR colonization OR “mycelial development” OR “mycelial production” OR “hypha* growth” OR “hypha* development”))	297	396	30
	Scopus	TITLE-ABS-KEY((Alternaria) AND (“Solanum lycopersicum” OR tomato) AND (“mycelial growth” OR invasion OR colonization OR “mycelial development” OR “mycelial production” OR “hypha* growth” OR “hypha* development”))	190		
	CAB Ab.	((mycelial growth or invasion or colonization or mycelial development or mycelial development or hypha$ growth or hypha$ development) and Alternaria and tomato).af.	249		
Incubation and latency periods	Web of Science	TS=((Alternaria) AND (“Solanum lycopersicum “OR tomato) AND (incubation OR “incubation period” OR “lesion onset” OR “lesion development” OR “latency” OR “laten* period”))	62	97	4
	Scopus	TITLE-ABS-KEY((Alternaria) AND (“Solanum lycopersicum” OR tomato) AND (incubation OR “incubation period” OR “lesion onset” OR “lesion development” OR “latency” OR “laten* period”))	37		
	CAB Ab.	((incubation or incubation period or lesion onset or lesion development or latency or latent period) and Alternaria and tomato).af.	65		
Secondary inoculum production	Web of Science	TS=((Alternaria) AND (“Solanum lycopersicum “OR tomato) AND (conidia OR spore*) AND (production OR development OR sporulation))	213	264	21
	Scopus	TITLE-ABS-KEY ((Alternaria) AND (“Solanum lycopersicum” OR tomato) AND (conidia OR spore*) AND (production OR development OR sporulation))	98		
	CAB Ab.	((production or development or sporulation) and conidia and Alternaria and tomato).af.	77		
Mycotoxin production	Web of Science	TS=((Alternaria) AND (“Solanum lycopersicum” OR tomato) AND (mycotoxin* OR toxic* OR alternariol OR AOH))	554	596	2
	Scopus	TITLE-ABS-KEY((Alternaria) AND (“Solanum lycopersicum” OR tomato) AND (mycotoxin* OR toxic* OR alternariol OR aoh))	242		
	CAB Ab.	((mycotoxin$ or toxic$ or alternariol or AOH) and Alternaria and tomato).af.	316		
Modeling	Web of Science	TS=((Alternaria) AND (“Solanum lycopersicum” OR tomato) AND (model* OR simulat* OR predict* OR forecast* OR prognos*))	221	237	28
	Scopus	TITLE-ABS-KEY((Alternaria) AND (“Solanum lycopersicum” OR tomato) AND (model* OR simulat* OR predict* OR forecast* OR prognos*))	111		
	CAB Ab.	((model$ or simulat$ or predict$ or forecast$ or prognos$) and Alternaria and tomato).af.	136		

The operator AND indicates that both terms must be present somewhere in the search field; the operator OR indicates that at least one term must be present in the search field. The high quotes (““) allow more than one word to be considered as a single item. The wildcard (*) allows the selection of multiple word endings.

The systematic literature review was performed in May 2023 with the digital bibliographical databases Web of Science (https://www.webofscience.com/accessed on May 17), Scopus (https://www.scopus.com/accessed on May 24), and CAB Abstract (https://www.cabdirect.org/cabdirect/search/accessed on May 24).

**Figure 1:**
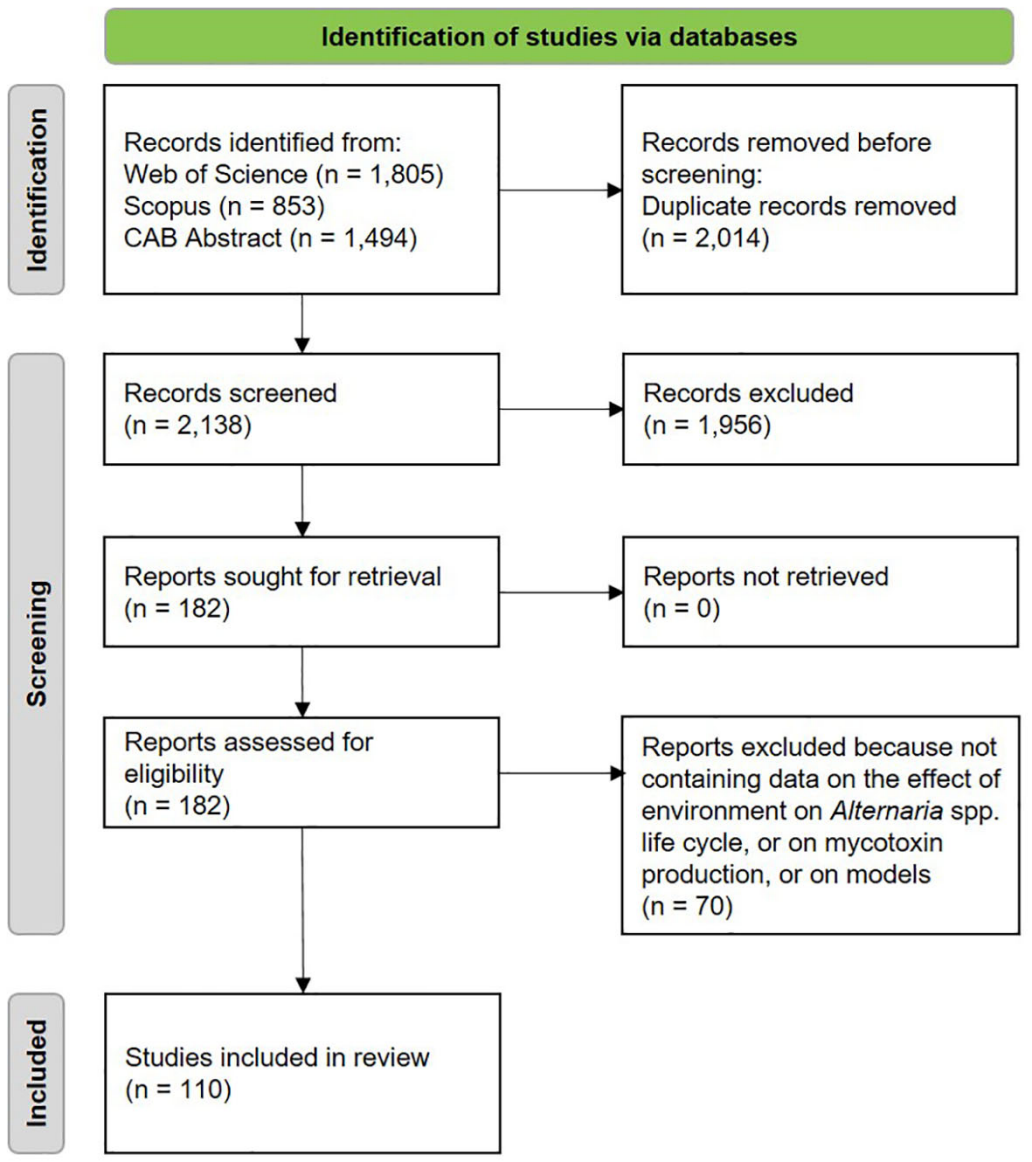
Schematic representation of the systematic literature review.

The data collection process was based on Biesbroek et al. (2013). Papers obtained from the first search were merged and duplicates were excluded. Papers were then screened by title and evaluated at the abstract level for relevance. The full texts of considered papers were reviewed to ensure their relevance for the fixed aims. Reference lists of the reviewed papers were checked for other studies meeting the eligibility criteria, yet they were not retrieved in the explored databases (**Figure 1**).

## 3 Results

### 3.1 Key characteristics of the selected papers

A total of 2,138 papers were obtained through the literature search. Among these, 1,542 papers concerned the effect of the environment on the main epidemiological components of *Alternaria* spp. life cycle, 596 were related to the production of mycotoxins, and 237 were retrieved through the specific query on epidemiological models. **Table 1** summarizes the number of retrieved papers from each database and the number of selected papers for each thematic block and sub-block.

A total of 80 papers were considered for the main epidemiological components of *Alternaria* spp. life cycle. The 80 papers referred to five *Alternaria* species, namely, *A. alternata*, *A. arborescens*, *A. solani*, *A. tenuissima*, and *A. tomato*. *Alternaria solani* and *A. alternata* were the most studied species, accounting for 59% and 24% of the studies, respectively. Most species were evaluated for mycelial growth (38%) and secondary inoculum production (26%). However, the literature search highlighted incomplete and fragmented information on epidemiological components, particularly for *A. arborescens*, *A. tenuissima*, and *A. tomato*.

Only two papers reported information on the effect of environmental factors on mycotoxin production in two *Alternaria* species, namely, *A. alternata* and *A. arborescens*.

In terms of epidemiological models, 28 papers reported information on their development, validation, or practical implementation. The models considered in these papers were built using empiric, machine learning, or mechanistic approaches, and were used to perform simulations or predict infection periods, disease progress, atmospheric spore content, the effect of climate change on *Alternaria* diseases, or to support the scheduling of fungicide application.

**Figure 2:**
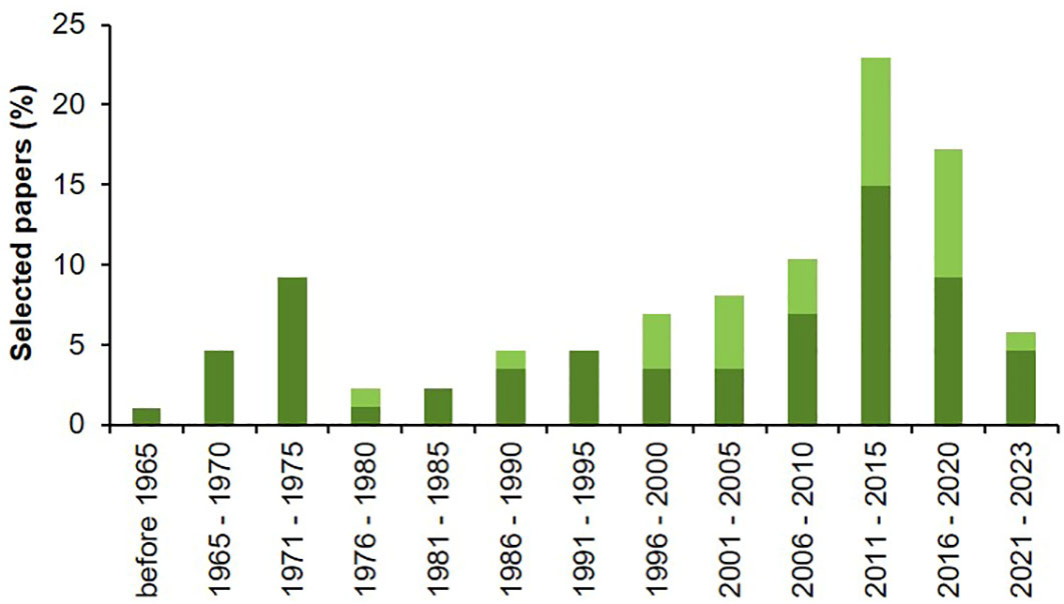
Percentage of selected papers published per 5-year periods that evaluated the influence of environmental conditions on main biological processes of *Alternaria* spp. affecting tomato (dark green) or include studies on modeling *Alternaria*-tomato pathosystem (light green).

Papers selected through the systematic literature search were published between 1936 and 2023 (**Figure 2**), with an increasing trend that reached its maximum in the 5-year period between 2011 and 2015, which accounted for 15% of the studies on biological processes and 8% of the studies on modeling. Despite studies on *Alternaria* spp. biology, ecology and epidemiology begun in the XX century, few models to address *Alternaria* diseases were proposed starting from the end of 1970s.

**Figure 3:**
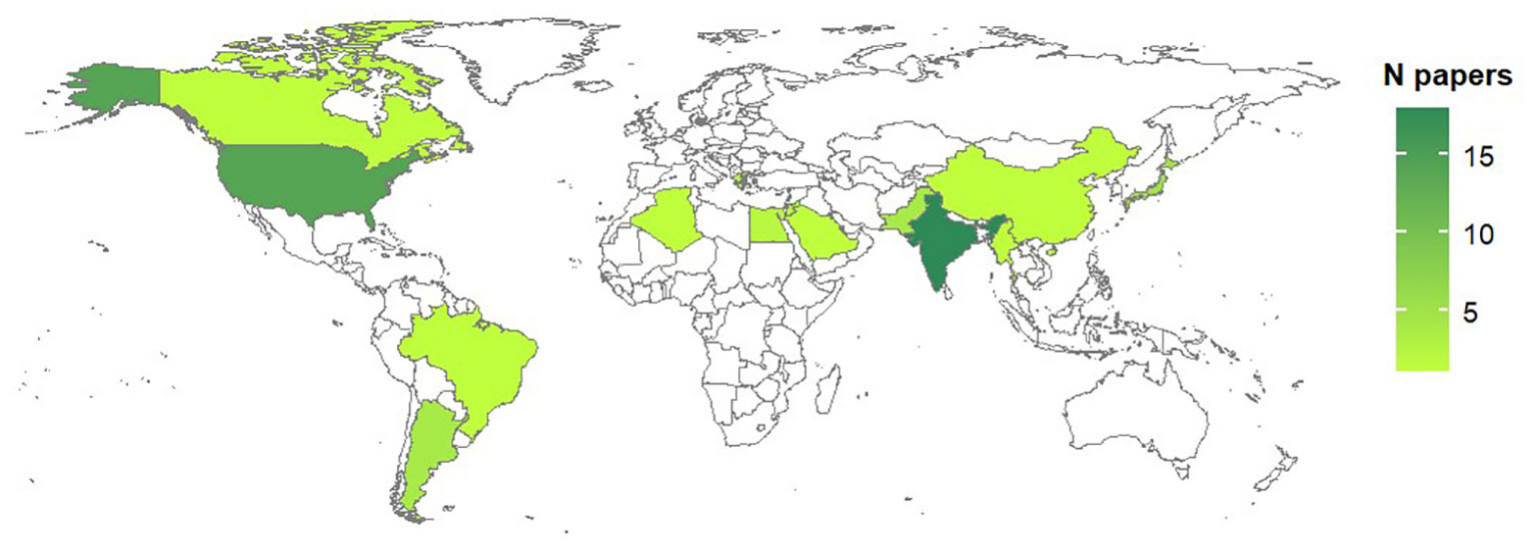
Country of origin of the studies on biological processes of *Alternaria* spp. affecting tomato. The total number of papers per country is shown as a color gradient, from light green (lower number of papers) to dark green (higher number of papers). Countries for which no papers have been found in the literature are white.

**Figure 4:**
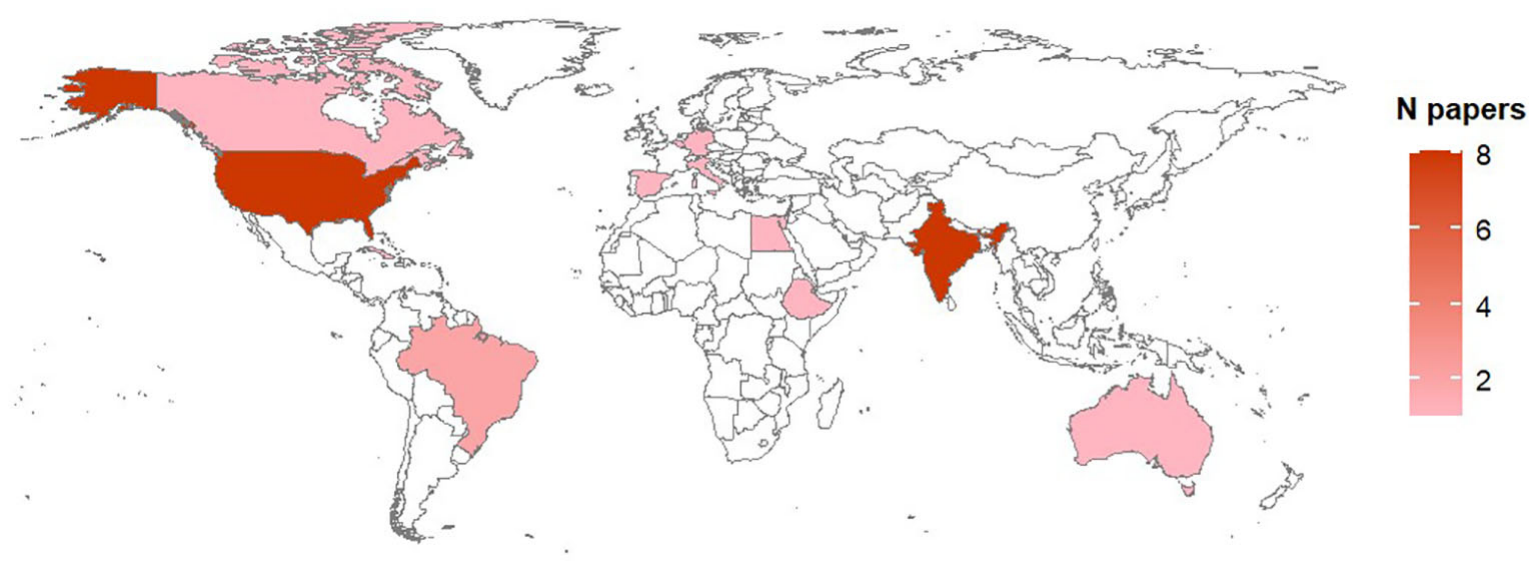
Country of origin of the studies on models available for *Alternaria* –tomato pathosystem. The total number of papers per country is shown as a color gradient, from light pink (lower number of papers) to dark pink (higher number of papers). Countries for which no papers have been found in the literature are white.

Studies on biological processes were performed in 14 countries over four continents (**Figure 3**); the majority of studies however were carried out in India (31% of studies) and USA (24%). Concerning published literature on modeling *Alternaria*-tomato pathosystem, studies were reported for 13 counties, mainly reflecting the distribution of studies on biological processes (**Figure 4**).

### 3.2 Main epidemiological components

#### 3.2.1 Primary inoculum sources

The number of retrieved studies on primary inoculum sources was limited and these studies only focused on *A. solani*. Basu (1971) reported that infected tomato tissue and associated soil samples contained viable conidia, mycelial fragments, and chlamydospores, which allowed *A. solani* to last at least seven months in soil, with or without plant debris. The pathogen survived equally well in plots where diseased plants were plowed before winter underground or were left aboveground until the following spring. According to Patterson (1991), conidia did not survive in soil for any significant length of time, and the long-term persistence of the pathogen in soil was achieved mainly by sources of chlamydospore inoculum differentiated from mycelium and conidia. Chlamydospores, in fact, can survive through adverse conditions, including soil and air temperature (T) ranging from −5°C to 33°C and −31°C to 28°C, respectively (Basu, 1971; Patterson, 1991). Patterson (1991) reported that chlamydospores survived 750 mm of rainfall in a season and two storms, causing 7 to 10 cm of snow and ice lasting 4–6 days. Primary infections caused by chlamydospores and mycelium lead to the development of foci to produce conidia that can initiate early blight epidemics, as well as collar and root rot (Jones et al., 1991; Patterson, 1991).

In mild locations *A. solani* can also survive from season to season on infected volunteer plants or other solanaceous hosts such as potato (*Solanum tuberosum* L.), eggplant (*S. melongena* L.), horsenettle (*S. carolinense* L.), and black nightshade (*S. nigrum* L.), which serve as a stock of primary inoculum propagules (Jones et al., 1991).

#### 3.2.2 Dispersal of conidia

Information on the environmental conditions favorable for conidial dispersal was included in four papers that considered *A. alternata* and *A. solani*. For both species, conidia dispersal was positively correlated with T and wind speed, and negatively correlated with relative humidity (RH), leading to a diurnal periodicity with a peak during the afternoon hours (Gottwald et al., 1997; Jambhulkar et al., 2016; Pearson and Hall, 1975). Both laboratory (Gottwald et al., 1997; Pearson and Hall, 1975) and field spore trapping (Jambhulkar et al., 2016) studies demonstrated that maximum conidial concentration was observed during periods of high mean T (25°C–30°C), high wind speed (5–10 km/h), and after a decrease in RH from 50% to 30%. An inverse correlation between low T and spore numbers was reported by Jambhulkar et al. (2016) in field and Pearson and Hall (1975) under controlled conditions. Jambhulkar et al. (2016) observed a low number of trapped conidia from January to March, with a sudden increase in April following a decrease in mean RH from 75% to 55%, an increase in the minimum T from 13°C to 21°C, and an increase in mean wind speed from 3.3 to 5.4 km/h. Pearson and Hall (1975) observed a drop in the number of trapped conidia after mid-September, linked to an increase in the number of hours with T ≤ 15°C.

Spore trapping studies on *A. solani* also revealed a positive relationship between the number of dispersed spores and rain. In controlled condition studies, Gottwald et al. (1997) observed that *A. solani* responded to rain showers by producing peaks of conidia release, while Waggoner and Horsfall (1969) reported that conidia can be dispersed in rain, with the equivalent of 6 mm of water removing approximately 99% of spores from leaflets.

#### 3.2.3 Germination of conidia

Nine papers investigated the environmental effects on conidial germination for *A. alternata*, *A. arborescens*, and *A. solani*. The species exhibited similar T requirements (**Table 2**), with germination occurring between 5°C and 32°C, and an optimum T range of 25°C–30°C (Kemmitt, 2002; Malathrakis, 1983; Pearson and Hall, 1975; Pose et al., 2009; Stevenson and Pennypacker, 1988; Thomidis et al., 2023; Vaquera et al., 2014; Waggoner and Parlange, 1974a, 1974b; Wang et al., 2002). Temperatures exceeding 32°C were tested for *A. alternata* and *A. solani*, which showed negligible germination up to 37°C and no germination at 40°C (Malathrakis, 1983; Thomidis et al., 2023; Waggoner and Parlange, 1974b).

**Table 2:** Temperature requirements of *Alternaria* spp. for biological processes considered for the literature search.

Biological process	Species	Temperature (°C)									
		0	5	10	15	20	25	30	35	40	45
Conidial germination	*A. alternata*										
	*A. arborescens*										
	*A. solani*										
	*A. tenuissima*										
	*A.tomato*										
Conidial infection	*A. alternata*										
	*A. arborescens*										
	*A. solani*										
	*A. tenuissima*										
	*A.tomato*										
Mycelial growth	*A. alternata*										
	*A. arborescens*										
	*A. solani*										
	*A. tenuissima*										
	*A.tomato*										
Symptoms development	*A. alternata*										
	*A. arborescens*										
	*A. solani*										
	*A. tenuissima*										
	*A.tomato*										
Sporulation	*A. alternata*										
	*A. arborescens*										
	*A. solani*										
	*A. tenuissima*										
	*A. tomato*										
Mycotoxin production	*A. alternata* (TeA)										
	*A. arborescens* (TeA)										
	*A. solani* (TeA)										
	*A. tenuissima* (TeA)										
	*A. tomato* (TeA)										
	*A. alternata* (AOH/AME)										
	*A. arborescens* (AOH/AME)										
	*A. solani* (AOH/AME)										
	*A. tenuissima* (AOH/AME)										
	*A. tomato* (AOH/AME)										

Colors indicate the response of the biological process to temperature as optimal (red), fair (orange), low (yellow), none (gray) based on experimental evidence. Empty cells indicate not tested conditions.

Conidia of *A. alternata* and *A. solani* germinated at RH ≥ 92% or water activity (a_w_) ≥ 0.9, but germination was higher when free water was available (Pearson and Hall, 1975; Pose et al., 2009; Stevenson and Pennypacker, 1988; Thomidis et al., 2023; Vaquera et al., 2014; Waggoner and Parlange, 1974c). The germination dynamic was affected by the combined effect of T and a_w_, RH, or the duration of wetness. In all the tested species, a shift from the optimum T or a reduction of RH or a_w_ increased the germination time. For example, 50% of *A. solani* conidia germinated within 1.2 h at 29°C and continuous wetness. However, a reduction in T caused a delay from 20 min (at 25°C) to about 20 h (at 4°C; Waggoner and Parlange, 1974a). Longer germination times were also observed in *A. alternata* and *A. arborescens* with a decline in RH or substrate a_w_ (Pearson and Hall, 1975; Pose et al., 2009; Vaquera et al., 2014). *Alternaria alternata* conidia began to germinate within 3 h over a large T range (20°C–37°C) in free water or at RH 100% (Malathrakis, 1983; Thomidis et al., 2023). However, Pearson and Hall (1975) observed that a slight reduction in RH from 100% to 98.5% induced a 3 h delay at the beginning of germination. Pose et al. (2009) reported a delay of about 5 days in the germination time of *A. alternata* by reducing a_w_ from 0.982 to 0.904. A similar negative relationship between the germination time and a_w_ was observed for *A. arborescens* by Vaquera et al. (2014), with a delay of 36 h at 20°C and a reduced a_w_ from 0.995 to 0.950.

The effect of light on conidial germination has only been reported for *A. solani*. Stevenson and Pennypacker (1988) observed maximum conidial germination in dark conditions, indicating that the inhibition of germination was positively correlated with the increase in light intensity and exposure. In particular, the authors reported an inhibition of up to 45% at light intensities and exposures comparable to sunny days during the northeastern US summer. Moreover, a stronger inhibitory effect was observed when UV wavelengths were included in the light spectrum.

#### 3.2.4 Infection by conidia

Specific experiments were conducted on the effect of environmental conditions and the plant growth stage on the conidial infection only for *A. alternata*, *A. solani*, and *A. tomato* in eight papers. All three species caused infection over a large range of T (10°C–30°C), with optimum conditions at 26°C, 27°C, and 30°C for *A. alternata*, *A. solani*, and *A. tomato*, respectively (Chohan et al., 2015; Malathrakis, 1983; Nightingale and Ramsey, 1936). The ability to cause infection at T < 10°C was reported for *A. solani*, which infected both leaves and fruits at 7°C (Nightingale and Ramsey, 1936) and 5°C (Chohan et al., 2015), but not for *A. tomato* (Nightingale and Ramsey, 1936). *Alternaria alternata* and *A. solani* also induced infection at 35°C (Chohan et al., 2015; Malathrakis, 1983). Temperatures lower than 10°C or higher than 30°C were not tested for *A. alternata* and *A. tomato*, respectively (**Table 2**).

Although *Alternaria* germ tubes can penetrate in leaf epidermis directly or enter through stomata under continuous wetness or alternating wet and dry periods (Kemmitt, 2002; Waggoner and Horsfall, 1969), the effect of wetness duration on the infection of tomato plants has been poorly studied, with laboratory tests only available for *A. solani* (Vloutoglou and Kalogerakis, 2000; Waggoner and Horsfall, 1969). Four hours of wetness were reported to be necessary for *A. solani* conidia to cause infection, while no symptoms were observed on tomato plants with wetness durations ≤ 3 h. Infection severity increased from 6 to 12 h, however, no further increase was observed with wetness durations of 24, 48, and 72 h (Vloutoglou and Kalogerakis, 2000; Waggoner and Horsfall, 1969).

Studies investigating the effect of light on infection are lacking for all species. Pearson and Hall (1975) conducted a field study to determine that UV light inhibits fruit infection by *A. alternata*.

In terms of the plant growth stage, the susceptibility of tomato to *A. alternata* and *A. solani* infection is directly related to aging and fruit ripeness (Nightingale and Ramsey, 1936; Pandey et al., 2003; Pearson and Hall, 1975; Qaryouti et al., 2012; Vloutoglou and Kalogerakis, 2000). The percentage of leaf area exhibiting symptoms and defoliation increased with the plant age (Pandey et al., 2003; Vloutoglou and Kalogerakis, 2000). The results of greenhouse and field studies reveal that the infection of green fruits usually remains limited, while infections on ripe fruits produce large and sunken lesions (Nightingale and Ramsey, 1936; Pearson and Hall, 1975; Qaryouti et al., 2012).

#### 3.2.5 Mycelial growth

Mycelial growth was studied *in vitro* conditions for all the *Alternaria* spp. considered in this review. Overall, 30 papers investigated the effect of medium composition, carbon and nitrogen sources, pH, and/or environmental conditions (T, a_w_, and RH). Most papers (20 out of 30) focused on *A. solani*, while *A. alternata* was the second most studied species (nine papers), with limited focus on *A. tomato* (two papers), *A. arborescens*, and *A. tenuissima* (one paper each).

All species grew at 10°C–30°C, with the optimum T ranging from 25°C to 30°C (**Table 2**; Akhtar et al., 1999; Alhussaen, 2012; Aziz et al., 1991; Bessadat et al., 2014; Chohan et al., 2015; Dushyant et al., 2014; Ilhe et al., 2017; Mahalakshmi et al., 2021; Malathrakis, 1983; Naik et al., 2010; Nightingale and Ramsey, 1936; Nikam et al., 2014; Pose et al., 2009; Rahmatzai et al., 2016; Roopa et al., 2016; Sinha and Alam, 2017; Somappa et al., 2013; Thomidis et al., 2023; Vaquera et al., 2014; Wang et al., 2002). T values out of this range have not been tested for *A. arborescens*. *Alternaria tenuissima* and *A. tomato* were also able to grow at 5°C and 35°C (Bessadat et al., 2014; Nightingale and Ramsey, 1936). *Alternaria solani* growth halted at T between 0°C and 5°C (Alhussaen, 2012; Rahmatzai et al., 2016; Nightingale and Ramsey, 1936; Thomidis et al., 2023) or ≥ 40°C (Alhussaen, 2012; Sinha and Alam, 2017; Thomidis et al., 2023). The isolate was observed to play a key role in *A. alternata* and *A. solani*; *A. alternata* grew at T ≤ 37°C (Malathrakis, 1983) and as low as 5°C in some isolates (Bessadat et al., 2014; Thomidis et al., 2023), but not in others (Akhtar et al., 1999; Malathrakis, 1983).

The effect of a_w_ on mycelial growth was investigated for *A. alternata* and *A. arborescens*. Pose et al. (2009) and Aziz et al. (1991) reported the growth of *A. alternata* to be supported at a_w_ ≥ 0.9, increasing proportionally with a_w_. Vaquera et al. (2014) reported *A. arborescens* growth for a_w_ ranging from 0.950 to 0.995, while lower a_w_ values were not tested.

Different RH regimes were tested for *A. solani*, with the highest RH range (90 – 100%) observed to optimize its growth (Naik et al., 2010; Somappa et al., 2013).

Over 15 synthetic media or tomato extracts were employed in mycelial growth experiments for *A. solani*, as solid or liquid substrates, primarily including agar-based or broth solutions of potato dextrose, oatmeal, cornmeal, vegetable juice (V8), and Czapek’s and Sabouraud’s media, and leaf, fruit, or stem extracts. The maximum growth was observed in either potato dextrose agar (PDA) or broth (PDB) (Chohan et al., 2015; Koley and Mahapatra, 2015; Mahalakshmi et al., 2021; Nikam et al., 2014; Nishant and Karuna, 2017; Roopa et al., 2016; Wang et al., 2002), or a mixture of these two substrates with tomato leaf or fruit extracts (Rex and Rajasekar, 2021). Several studies reported mycelial growth comparable to PDA and PDB in agar or broth of Czapek’s Dox media (Nagesh et al., 2021; Somappa et al., 2013), Sabouraud’s media (Naik et al., 2010; Rahmatzai et al., 2016), and V8 (Kumar et al., 2008). In terms of carbon sources, glucose (Dhal et al., 2014; Dushyant et al., 2014; Rex and Rajasekar, 2021; Wang et al., 2002) and starch (Nikam et al., 2014) supported maximum mycelial growth, while stunted mycelial colonies were observed on media mixed with xylose (Nikam et al., 2014) or citric and lactic acids (Dhal et al., 2014). Several amino acids and nitrogen sources were identified to boost colony growth, including threonine (Dhal et al., 2014), asparagine (Dushyant et al., 2014), tyrosine (Wang et al., 2002), and nitrate of potassium (Nikam et al., 2014) and ammonium (Rex and Rajasekar, 2021). *Alternaria solani* was observed to grow within the pH range of 2–12 (Wang et al., 2002), with a maximum growth at 6–7 pH (Ahmad et al., 2014; Alhussaen, 2012; Chohan et al., 2015; Dushyant et al., 2014; Mahalakshmi et al., 2021; Nikam et al., 2014; Sinha and Alam, 2017; Wang et al., 2002).

*Alternaria alternata*, *A. tenuissima*, and *A. tomato* grow well on potato-based media, vegetable or tomato juice agar, and Czapek’s agar (Abdel-Mallek et al., 1995; Bessadat et al., 2014; Hylin et al., 1970; Ilhe et al., 2017). Numerous carbon and nitrogen sources are reported to be suitable for *A. alternata* and *A. tenuissima*, including glucose, mannitol, and citric acid, and sodium nitrate, potassium nitrate, and asparagine, respectively (Bessadat et al., 2014). Similar to *A. solani*, mycelial growth for both species was supported under a wide pH range (4–10, the entire tested range), with an optimum pH range of 6–8 (Bessadat et al., 2014).

No additional information is reported in the literature for *A. arborescens* or *A. tomato*.

#### 3.2.6 Development of symptoms

Fragmented information was found on the length of the incubation period (time from infection until the onset of symptoms). As a general requirement for *A. solani*, Kemmitt (2002) and Waggoner and Horsfall (1969) reported symptoms on the foliage to become visible within 5–7 days. Lesions remained as small spots in dry leaves, which rapidly grew when leaves were moist. On fruits, *A. solani* symptoms appeared within 2 days at 27°C, and the incubation period increased when T shifted from this optimum. At 7°C, the symptoms did not appear before the tenth day (Nightingale and Ramsey, 1936). The same temperature-dependent behavior was observed in *A. alternata* and *A. tomato* (**Table 2**). Five to 6 days were required for disease onset at T between 10°C and 16°C for both species, and for *A. alternata* at 35°C (Malathrakis, 1983; Nightingale and Ramsey, 1936). After the occurrence of infection under favorable environmental conditions, *A. tomato* was able to develop symptoms at T between 0°C and 5°C, or 7°C in 8 days, yet lesions remained small and shallow (Nightingale and Ramsey, 1936).

#### 3.2.7 Sporulation

The effects of environmental factors (T, light, and RH) and media composition on conidial production were evaluated for *A. alternata*, *A. solani*, *A. tenuissima*, and *A. tomato* in 21 papers.

All four species sporulated between 10°C and 30°C, exhibiting optimal T at around 20°C in *A. tomato* and *A. tenuissima*, or between 25°C and 30°C in *A. alternata* and *A. solani* (**Table 2**; Bessadat et al., 2014; Naik et al., 2010; Pearson and Hall, 1975; Singh, 1967; Sinha and Alam, 2017; Somappa et al., 2013; Waggoner and Horsfall, 1969). In these species, however, a marked reduction in sporulation was observed at T ≤ 15°C or ≥ 33°C (Pearson and Hall, 1975; Waggoner and Horsfall, 1969). Temperatures less than 10°C were tested for *A. tenuissima* and *A. alternata*. The former was unable to produce conidia at 5°C (Bessadat et al., 2014) and the latter poorly sporulated at 9°C, 6°C, or 5°C (Bessadat et al., 2014; Pearson and Hall, 1975). *A. tenuissima* and *A. alternata* were also able to sporulate at T as high as 35°C–36°C (Bessadat et al., 2014; Pearson and Hall, 1975), while *A. tomato* did not produce conidia at T ≥ 31°C (Kumagai, 1989). Contrasting data were collected from the literature on the maximum T for the sporulation of *A. solani*. Singh (1967) and Waggoner and Horsfall (1969) reported no or negligible conidial production at T ≥ 27°C, and most of the mycelial fragments failed to differentiate into conidiophores at 32°C and 37°C. In contrast, some isolates exhibited reasonable sporulation between 35°C and 44°C (Sinha and Alam, 2017; Somappa et al., 2013).

Studies on *A. alternata* and *A. solani* showed that sporulation was enhanced by free water in the substrata and high RH, with optimum activity at RH between 90% and 100% (Naik et al., 2010; Somappa et al., 2013; Waggoner and Horsfall, 1969). *Alternaria solani*, however, was able to produce a large quantity of conidia at RH 60% (Somappa et al., 2013), while *A. alternata* did not produce spores at RH ≤ 65% (Naik et al., 2010).

Although light was the most studied environmental factor (11 out of 21 papers), tests were only conducted on *A. solani* and *A. tomato*. Both species exhibited good sporulation in alternate light–dark conditions and conidia production was induced following exposure to UV radiation (Aragaki et al., 1973; Kumagai, 1978, 1983, 1986, 1989; Kumagai and Oda, 1969; Naik et al., 2010; Rodrigues et al., 2010; Wang et al., 2002; Yadav et al., 2015; Waggoner and Horsfall, 1969). Waggoner and Horsfall (1969) reported that exposure to light for a few minutes triggered the development of conidiophores, which were able to grow in both dark and light conditions.

*Alternaria* spp. was observed to sporulate on various substrata in tomato fields, including leaf and fruit lesions, senescent and non-living plant material, and healthy leaflets covered by aphid honeydew (Pearson and Hall, 1975). In controlled conditions, the substrata observed to support abundant spore production in all the studied species were potato-based media (e.g., PDA) or host extracts, as well as synthetic media such as Sabouraud’s agar (Bessadat et al., 2014; Hylin et al., 1970; Ilhe et al., 2017; Khin et al., 2007; Naik et al., 2010; Nishant and Karuna, 2017; Rodrigues et al., 2010; Somappa et al., 2013; Wang et al., 2002). Nishant and Karuna (2017) and Somappa et al. (2013) reported the addition of CaCO_3_ to media to boost spore production in *A. solani*. In terms of substrate pH, *Aternaria alternata*, *A. solani*, and *A. tenuissma* were observed to sporulate at the pH range 3–10, with favorable production between 4 and 7.5 pH (Ahmad et al., 2014; Bessadat et al., 2014; Sinha and Alam, 2017). The effect of different carbon and nitrogen sources was investigated by Bessadat et al. (2014) on *A. alternata* and *A. tenuissima*. While maximum sporulation was recorded when the substrate contained sodium nitrate or potassium nitrate, limited effects were observed by changing the carbon source.

### 3.3 Mycotoxin production

TeA, AOH, and AME are commonly detected in fresh tomatoes collected from fields (Sanzani et al., 2019; Qin et al., 2022). However, minimal information is available regarding the different capabilities of *Alternaria* species to produce the different types of *Alternaria* toxins. TeA, AOH, and AME, the main contaminants of food commodities, are all produced by *A. alternata* (Scott, 2001, Ostry, 2008, Logrieco et al., 2009; Bellotti et al., 2023). However, *in vitro* studies on *A. tenuissima* (Bellotti et al., 2023) and *A. arborescens* (Vaquera et al., 2016) underlined their ability to produce these toxins too. In terms of *A. solani*, not all strains are able to produce TeA (Bellotti et al., 2023).

*A. alternata* and *A. arborescens* are the only species included in ecological studies that consider the effect of T and a_w_ on the production of toxins.

The production of TeA by *A. alternata* was maximized at 0.982 a_w_ and 21°C after 28 days on Tomato Pulp Agar. TeA was reduced by 62% at 0.954 a_w_, while at 0.922 a_w_, small amounts were only detected at 21°C. However, 21°C was the optimal T for TeA production, irrespective of the a_w_ value, while 6°C was the lower limit (**Table 2**). The toxin was produced only at a_w_ ≥ 0.982 (Pose et al., 2010). For *A. arborescens*, the production of TeA was maximized at 0.975 and 25°C after 30 days and at 0.950 and 30°C after 21 days (Vaquera et al., 2016).

Optimal conditions to produce AOH by *A. alternata* were reported as 0.954 a_w_ at 21°C after 28 days. As a_w_ increased, the amount of AOH decreased at various degrees depending on T (**Table 2**). As with TeA, the optimal T for AOH production was 21°C, independent of the a_w_ value (Pose et al., 2010). In terms of *A. arborescens*, the maximum production of AOH was observed at 30°C and a_w_ at 0.975 under a longer incubation time (40 days) (Vaquera et al., 2016). The optimal conditions to produce the monomethyl ether (AME) are similar to those observed for AOH production for both *A. alternata* and *A. arborescens*.

Information on the ecological parameters influencing the production of *Alternaria* toxins by other *Alternaria* species in tomato is lacking in the literature, with no studies conducting trials on fresh tomato nor experiments on synthetic medium based on tomato pulp. Moreover, the consideration of ecological parameters is limited, particularly for a_w,_ which was only considered at levels higher than 0.95.

### 3.4 Modeling support for disease management

A total of 28 papers presented epidemiological models developed for *Alternaria* diseases, with all models focusing on the early blight on leaves and fruit rots. More than half of these papers (64%) proposed new epidemiological models developed *ex novo* through the statistical analysis of disease data collected in the field. The rest of the papers (36%) focused on the validation (Cahn et al., 1997) and practical implementation of existing models, with trials performed to evaluate the ability of models to guide tactical decisions for disease management (Keinath et al., 1996; Louws et al., 1996; Mills et al., 2002; Minchinton et al., 2006) or to compare different models (Batista et al., 2006; Cowgill et al., 2005; Tietjen et al., 2001), or simulations under different climate change scenarios (Van de Perre et al., 2015; Van der Fels-Klerx et al., 2016).

Most of the developed models aim to predict *Alternaria* occurrence in terms of infection periods (Madden et al., 1978; Srivastava et al., 1989; Waggoner and Horsfall, 1969), disease progress (Devi et al., 2017; Gupta et al., 2020; Laurindo et al., 2017; Paul et al., 2019; Peña et al., 2012; Ruth et al., 2016; Saha and Das, 2014a, 2014b; 2015a, 2015b; Tadelo et al., 2022), and atmospheric spore content (Astray et al., 2010). Among these, only one model was developed based on the *Alternaria* life cycle using a mechanistic approach, namely, EPIDEM (Waggoner and Horsfall, 1969). This model was developed based on biological data of *A. solani* and incorporates mathematical relationships that modulate the effect of environmental data (T, RH, wind speed, rainfall, and sunshine) on the following fungal stages: formation of conidiophores and spores; spore dispersal by wind or rain; deposition; germination; penetration; incubation; and lesion growth.

The other models follow an empirical approach by relating weather data to *Alternaria* occurrence using statistical analysis and regression functions or exploiting neural network models. Most of these models were not validated against independent data, only with internal validation, with coefficient of determination (R^2^) varying from 0.21 to 0.98 (Astray et al., 2010; Devi et al., 2017; Gupta et al., 2020; Laurindo et al., 2017; Paul et al., 2019; Peña et al., 2012; Ruth et al., 2016; Saha and Das, 2014a, 2014b; 2015a, 2015b). Attention was also paid to develop empirical models for the identification of risk periods and the consequent scheduling of fungicides. This is the case of FAST model (Madden et al., 1978), which combines two empirically developed models to identify periods of favorable environmental conditions to early blight and provide a schedule for efficient fungicide application. FAST incorporates functions to determine daily disease severity (S) and daily severity-rating values (R) that are combined in rules used to determine periods suitable for fungicide spraying. Several factors other than environmental parameters have been integrated into this model, including host resistance (Shuman and Christ, 2005). FAST has also been adopted and modified for the release and evaluation of other models like TOMCAST, CU-CAST, NJ-CAST, which were compared in several field studies. Overall, the use of these models resulted in a reduction in the number of fungicide applications compared to the common strategy based on weekly scheduling, while ensuring disease control. For instance, Tietjen et al. (2001) showed in a 2-year study in New Jersey that FAST, CU-CAST, and TOMCAST reduced early blight severity compared to the untreated control with as few as three sprays (vs. 11 applications of weekly schedule). These results were consistent with those of the other studies carried out in the USA (Batista et al., 2006; Cahn et al., 1997; Cowgill et al., 2005; Keinath et al., 1996; Louws et al., 1996; Mills et al., 2002) or Australia (Minchinton et al., 2006); no information was however retrieved for their application in other countries.

Only two papers presented new simulation models, one concerning the effect of climate change on early blight prediction (Hassan et al., 2019) and the other linking the growth dynamics of tomato to early blight epidemics (Chelal et al., 2015). Performances of both models were evaluated for their ability to represent the datasets used in their development, with R^2^ of 0.83 and 0.99, respectively. No validation with independent data was however performed.

## 4 Discussion

This review considers several important aspects of the *Alternaria* spp. life cycle and mycotoxin production in tomato cropping systems, with the aim of collecting available information for the development of a predictive, mechanistic model that considers multiple *Alternaria* species affecting tomato. Published species–specific models were also analyzed to evaluate their possible exploitation and incorporation into such a new model.

Most of the published, predictive models were built with an empirical approach that relates weather data to *Alternaria* occurrence using statistical or machine learning methods. These models incorporate single components of the pathogen life cycle, mainly infection, or consider the progress of the disease as weather-dependent only, irrespective of fungal biology. Furthermore, many of these models have never been validated against independent data. More specifically, model predictions have not been compared with real-world observations different from those used for model development. Only a few models, such as FAST, TOMCAST, CU-CAST, and NJ-CAST, were evaluated for their ability to predict the occurrence of early blight and support the scheduling of fungicide applications in real epidemics. Model performances, however, differed both among and within studies (Batista et al., 2006; Cowgill et al., 2005; Keinath et al., 1996), showing a strong dependency on the dataset used for model development and poor predictive ability beyond dataset boundaries. Limits of empirical models have been reported for several pathosystems, and the need for recalibration was reported when these models are applied in different epidemiological conditions (Caffi et al., 2007; Narouei-Khandan et al., 2020). In contrast, mechanistic models have the potential ability of correct predictions in a wide range of agricultural contexts due to their knowledge-based structure that incorporates the description and quantification of synchronous interactions between the pathogen, the host, and the environment governing the disease development (Rossi et al., 2010; De Wolf and Isard, 2007). The development of mechanistic models, therefore, implies the extensive collection of information on fungal biology, ecology, and epidemiology, as influenced by environmental factors and pathogen–host interactions.

This systematic review enables a comprehensive overview and quantitative analysis of literature by reducing search errors and bias and facilitating the communication of findings in an easy and user-friendly format (Madden and Paul, 2011; Candel, 2014). In this study, *Alternaria* spp. life cycle was considered as a continuous sequence of biological events and consequently divided into seven major biological processes to search for in the literature. The available knowledge was extracted from a total of 110 papers (from an initial number of 2,138) that considered five *Alternaria* species, both well-known to cause disease in tomato, namely, *A. solani*, *A. alternata*, and *A. tomato*, or the recently identified *A. arborescens* and *A. tenuissima* (e.g., Schmey et al., 2024; Bessadat et al., 2017; Andersen et al., 2015; Waggoner and Horsfall, 1969; Nightingale and Ramsey, 1936). Inclusion and exclusion criteria applied to retrieved articles allowed us to focus on the research on the *Alternaria–*tomato pathosystem and exclude knowledge bias due to pathogen interaction with other hosts that could lead to imprecise modeling of the *Alternaria* spp. disease cycle in tomato. Great variability in pathogenic pathways of polyphagous species has been reported, for example, *Botrytis cinerea* (e.g., van Kan, 2005; Williamson et al., 2007) and *Colletotrichum acutatum sensu lato* (Peres et al., 2005; Salotti et al., 2022; 2023), resulting in the need of developing host-specific models. To our knowledge, this is the first research that summarizes the available published information to evaluate the possibility of developing a mechanistic, weather-driven model for the prediction of *Alternaria* diseases in tomato. Furthermore, available reviews on *Alternaria* spp. do not focus on tomato, or whether tomato is considered, the review provide food for thoughts on several aspects, from phylogenetic to disease control, but no specific information for single biological processes. An overview of published models is a novelty, too, and lay the foundation for developing new tools for disease control.

Although the genus *Alternaria* has been investigated since the beginning of the last century, important gaps in understanding the effect of the environment on its life cycle remain for several species. Limited and inconsistent information was retrieved for primary inoculum sources and conidial dispersal. Although every step of the disease cycle is theoretically important, it is often not necessary to model all to develop reliable prediction tools (De Wolf and Isard, 2007). Modeling the availability of primary inoculum may increase the predictive ability of the models. However, for diseases caused by pathogens that survive locally within the debris of a previous crop, it is possible to assume that inoculum is not a limiting factor, and we can thus focus on modeling the pathogenic stages of the disease (De Wolf and Isard, 2007). The relationships of temperature and moisture to disease development and pathogen reproduction may therefore represent the basis for the development of a process-based model for the *Alternaria–*tomato pathosystem.

Scholars however have paid much more attention to the effects of temperature on mycelial growth than on other biological processes. Numerous missing data were highlighted, particularly for *A. arborescens* and *A. tenuissima*, whereby specific studies on conidial germination, infection, and onset of symptoms were not available. Missing information on the effect of temperature on *A. arborescens* sporulation was also observed. Furthermore, the studied temperatures were frequently restricted to the 10°C–35°C range, leading to a lack of information on the minimum and maximum temperatures able to support the considered biological processes. Overall, consistency in the optimum temperatures (25°C–30°C) among *Alternaria* species was observed, apart from sporulation with higher temperatures for *A. alternata* and *A. solani* (25°C–30°C) compared to *A. tenuissima* and *A. tomato* (20°C). The temperature-related response however differed between species when shifting to intermediate temperature, particularly for infection and sporulation.

Fragmented information was also retrieved for the effect of moisture (expressed as RH, water activity, or wetness) on the considered biological processes. This was also true for the most studied species, namely, *A. alternata* and *A. solani*. For example, the effect of wetness duration on germination was studied for both species, yet only Vloutoglou and Kalogerakis (2000) and Waggoner and Horsfall (1969) provided quantitative data on *A. solani* infection.

Along with disease prediction, the risk of mycotoxin production has been modeled for several mycotoxigenic fungi to meet reglementary and market needs, and to secure healthy foods and feeds in an integrated crop protection scenario under climate change (Battilani, 2016). Given the increasing interest in *Alternaria* mycotoxins in research programs and the regulatory framework of tomato production chains worldwide, more published studies are required on the environmental factors that trigger mycotoxin production. Information was collected only for the effect of temperature and water activity on *A. alternata* (Pose et al., 2010) and *A. arborescens* (Vaquera et al., 2016). These studies were performed *in vitro* on artificial media and considered a limited number of conditions (less than 15 combinations of temperature and water activity); no experiments collected data on infected tomatoes.

Overall, the insufficient database obtained through our literature review highlights the knowledge gaps on the environmental requirements for *Alternaria* spp. pathogenic biological processes and mycotoxin production, which are the foundation for the development of mechanistic models. Collected information can be exploited to develop temperature- and wetness-dependent functions for key steps of *A. solani* or *A. alternata* life cycle, as infection, symptoms development, and sporulation, and then incorporated into a simple framework for disease progress prediction. For the other species, however, further ecological and epidemiological studies are needed to relate the pathogen response to environmental changes. Moreover, to increase model’s predictive ability and produce a risk for mycotoxin contamination, primary inoculum origin, spore dispersal, and mycotoxin production should be deeply investigated to include these compartments into the model structure. Modelers often face the problem of defective knowledge and a lack or ambiguity of information related to the pathosystem (Rossi et al., 2010). Deficiencies have often been solved by making assumptions about processes that are, at least temporarily, included in a model (Wainwright and Mulligan, 2004). Assumptions originate from biologically plausible values and empirical probability distributions taken from related species and similar pathosystems (Wainwright and Mulligan, 2004; Rossi et al., 2010). These assumptions, however, could lead to less accurate predictions and should be carefully considered and improved while addressing the knowledge gaps.

This review presents the information available for the modeling of *Alternaria* diseases in tomato, highlights bottlenecks for the development of such mechanistic models, and aids researchers in identifying the necessary research required on the biology and epidemiology of *Alternaria* species.
